# 

**Published:** 1900-08-11

**Authors:** 


### NOTICE TO CORRESPONDENTS.

**All MS., Letters, Books for Review, and other matters intended for the Editor should be addressed The Editor, "The Hospital," The Hospital Building, 28 & 29, Southampton Street, Strand, W.C.**

**The Editor cannot undertake to return rejected MS., even when accompanied by stamped directed envelope.**

**Notice to Advertisers and Subscribers.**

All Advertisements, Orders for Copies of The Hospital, and Business Communications should be addressed to **THE MANAGER (Not to the Editor)**, The Hospital, The Hospital Building, 28 & 29, Southampton Street, Strand, London, W.C.

The Cost of Subscription, post free, is as follows:—Per week, 2½d.; per quarter, 2s. 8d.; per half-year, 5s. 3d.; per annum, 10s. 6d. Foreign:—Per annum, 15s. 2d., payable, in all cases, in advance.

**NOTICE.—Vol. XXVII. of "The Hospital" (October, 1899, to April, 1900), in handsome cloth binding, is now on sale at the Office of this Journal, price 7s. 6d. Cases for binding the Numbers contained in this Volume 1s. 6d. Index to Vol. XXVII., 2½d., post free. The Monthly Part for August is now ready. Price 6d. by post 8d.**

### BOOKS RECEIVED.

G. W. Bacon and Co.

"Bacon's New Large-Print Map of China."

Cassell and Co.

"Tropical Diseases." By Patrick Manson, C.M.G., M.D., LL.D. (Aberdeen).

**Periodicals Received.**—Boys' Own Paper, Leisure Hour, Medical Press, Leeds Hospital Magazine, Nursing Notes, Nature Notes, Sunday at Home, Monthly Homœopathic Review, Indian Review, Windsor Magazine, Guy's Hospital Gazette, Sanitary Record, Literary Digest, Charity Organisation Review.

### THE STUDENT OF MEDICINE.

### APPOINTMENTS.

E. Binney, M.B., appointed Surgeon to the Children's Hospital, Glebe, Sydney. E. De Marco, M.D., appointed Government Medical Officer and Public Vaccinator at Coolah, New South Wales. R. Emmett, L.R.C.P.Lond., M.R.C.S.Eng., appointed Certifying Factory Surgeon for the County Borough of Portsmouth and the Civil Parish of Cosham. H. C. Ensor, M.R.C.S., appointed Ophthalmic Surgeon to the Cardiff Provident Dispensary and to the Cardiff Infirmary. J. F. Flashman, M.D., appointed Pathologist in the Lunacy Department, New South Wales. H. H. Fleming, M.D., appointed Health Officer for the Shire of Kara Kara, Victoria. H. C. Fox, M.R.C.S.Eng., L.S.A., appointed Assistant Registrar to the London Throat, Nose, and Ear Hospital. R. A. Fox, M.B., appointed Junior Medical Officer in the Lunacy Department, New South Wales. J. Gott, L.S.A., appcinted Certifying Factory Surgeon for the Ringwood Rural District. T. F. Hanly, L.R.C.P., L.R.C.S.Edin., appointed Certifying Factory Surgeon for the Gillingham District of Dorset. A. W. C. Herbert, L.S.A., appointed Certifying Factory Surgeon for the Southwold District. J. C. Kuipers, M.D., appointed Health Officer at Eidsvold, Queensland. A. Miles, F.R.C.S.Edin., appointed Lecturer on Surgery in the Medical College for Women, Edinburgh. R. G. Naylor, L.R.C.P., appointed Health Officer for the Borough of Browns and Scarsdale and Borough of Smythesdale. J. A. Nolan, M.R.C.S.Eng., L.R.C.P.Edin., appointed District Surgeon, Camperdown, Natal, South Africa. W. H. Orchard, M.B., appointed Health Officer and Public Vaccinator for Port Fairy, Victoria. D. E. Pereira, L.R.C.P., L.R.C.S.Edin., L.F.P.S.Glasg., appointed House Surgeon to the Italian Hospital, Queen Square, W.C. A. T. Rimell, L.R.C.P.Edin., M.R.C.S.Eng., appointed Certifying Factory Surgeon for the Long Sutton District of Lincolnshire. J. P. Scatchard, M.B., B.S.Lond., appointed Certifying Factory Surgeon for the Tadcaster District of the West Riding of Yorkshire. E. G. Smith, L.R.C.P.I., L.S.A., appointed Certifying Factory Surgeon for the Walton-on-Naze District of Essex.

